# Knee osteoarthritis patient perspectives of their care in an australian private physiotherapy setting: a qualitative exploratory interview study

**DOI:** 10.1186/s12891-023-06692-4

**Published:** 2023-07-11

**Authors:** Jeanette M Thom, Sarah Dennis, Kathryn A Gibson, Rebecca Livings, Kathryn Mills, Siobhan M Schabrun, Hans Sun, Justine M Naylor

**Affiliations:** 1 School of Health Sciences and Sydney Musculoskeletal Health, Faculty of Medicine and Health, The University of Sydney, Sydney, Australia; 2 Faculty of Medicine and Health, UNSW, Sydney, Australia; 3 Ingham Institute of Applied Medical Research, Liverpool, Australia; 4 Rheumatology Department, Liverpool Hospital, Sydney, Australia; 5 Eli Lilly Australia Pty Ltd., Sydney, Australia; 6 Neuroscience Research Australia, UNSW, Sydney, Australia; 7 Department of Health Sciences, Macquarie University, Sydney, Australia; 8 School of Physical Therapy, University of Western Ontario, London, ON Canada; 9 The Gray Centre for Mobility and Activity, Parkwood Institute of Rehabilitation, London, ON Canada; 10 Whitlam Orthopaedic Research Centre, Orthopaedic Department, Liverpool Hospital, Sydney, Australia

**Keywords:** Knee osteoarthritis, Physiotherapy, Physical therapy, Guideline-based care

## Abstract

**Purpose:**

This study aimed to understand perceptions that knee osteoarthritis patients have regarding their experiences of guideline-based recommendations within their care received from physiotherapists in private practice.

**Methods:**

A qualitative semi-structured interview study nested within a larger trial auditing care provided by physiotherapists. Recruited adults ≥ 45 years with knee osteoarthritis across nine primary care physiotherapy practices. Interview questions were anchored around the core elements recommended in guidelines for the management of knee osteoarthritis and patient perceptions of these were analysed using both content and thematic qualitative analysis approaches. Patient satisfaction with care received was asked at the time of interview.

**Results:**

Twenty-six patients volunteered for the study (mean 60 years, 58% female). Analysis identified that physiotherapists focused on treating symptoms through quadriceps strengthening exercises, which patients found to be effective, though focussed less on other aspects of evidenced-based care. Patient’s perceived treatment to be effective in relieving pain and enabling them to stay active and they appreciated the positive role that their physiotherapist provided in alleviating their concerns. Overall, patients were satisfied with their physiotherapy care but would have liked more specific osteoarthritis education and longer-term management.

**Conclusion:**

The description of the physiotherapy-related care received by people with knee osteoarthritis aligns with guideline recommendations, though mainly for strength-related exercise prescription. Despite some perceived shortfalls in care, patients do appear to be satisfied. However, improvements in patient outcomes may be possible if more elements of guideline-base care are regularly provided, including enhancing osteoarthritis education and fostering behaviour change.

**Trial registration:**

ACTRN12620000188932.

**Supplementary Information:**

The online version contains supplementary material available at 10.1186/s12891-023-06692-4.

## Introduction

The prevalence of osteoarthritis is increasing globally, with a strong link to an ageing population and higher obesity levels [1, 2], ultimately contributing to greater need and cost of knee and hip replacement surgeries [1]. However, joint replacement is only appropriate for severe symptomatic joint disease with late-stage structural changes, thus, is not indicated for approximately 80% of people with osteoarthritis [3]. For those with early or less severe disease, a multidisciplinary approach that facilitates the preservation of joint structure and societal participation is both desirable and recommended.

There is considerable evidence for the benefits of non-surgical management of knee osteoarthritis via targeted multidisciplinary programs, with several guidelines proposing similar recommendations [4–8]. Guidelines consistently include education, land-based exercise and weight management (if indicated) as part of first line care which can be delivered by physiotherapists [9–12]. This early conservative management has been shown to decrease the societal burden, improve the quality of life of people with osteoarthritis and prevent many from requiring joint replacement surgery and hence reduce overall health system costs [3, 13].

The challenge, however, is to incorporate this evidence-based high value allied health care into practice. Currently, community-based osteoarthritis care has been found to be sub-optimal and referral to physiotherapy is low [14–16]. Clinician reliance on low-value care such as over-reliance on pharmacological interventions [17] and their failure to address or support weight management [18–20] is evident within the healthcare system. In order to align care to that recommended by clinical guidelines, there is a need to first understand the current systems [21].

Physiotherapists, especially in primary care, are key stakeholders in the healthcare of people with knee osteoarthritis at all stages, along with general practitioners who are the primary point of care [22]. However, the referral rates in Australia from general practitioners to physiotherapists for musculoskeletal issues, including osteoarthritis, remains low [23–25]. Once referred, physiotherapists may still focus on only certain aspects of evidence-based care, such as exercise management to restore functional ability, with lower incorporation of other aspects e.g. osteoarthritis education, psychosocial consideration and referral for weight loss [26, 27]. This is highlighted in our previous audit of physiotherapy care [28], though we currently have limited understanding of what evidence-based care physiotherapists provide from the patient’s perspective. Physiotherapists may lack knowledge or be uncertain how to incorporate the guidelines, for example the complexity of weight management into their treatment [16, 29]. A further barrier may be the patients themselves, with expectations that physiotherapy treatment is confined to exercise-based care and their biomechanical beliefs of osteoarthritis [30], as well as some patients’ uncertainty of care outcomes, including from lack of support/information from health professionals, their lack of motivation to exercise or belief that their osteoarthritis symptoms will improve [31].

As part of a larger study designed to test the feasibility of implementing guideline-based care for knee osteoarthritis in private physiotherapy practices across metropolitan Sydney [28, 32], we conducted semi structured interviews with patients with knee osteoarthritis to understand perceptions held by them regarding their experiences of care received from physiotherapists in private practice. Specifically, we aimed to determine if (1) patient reports of care received aligned with guideline-based recommendations for physiotherapy management, (2) whether they were satisfied with the care provided, and (3) to explore patient perceptions on the care they received.

## Materials and methods

### Design and setting

A qualitative study was used to capture current physiotherapy management of patients with knee osteoarthritis attending private practice clinics in Sydney. These data form part of a larger study, with the full protocol published [32] and registered at ClinicalTrials (ACTRN12620000188932, ACTRN12620000218998). Ethical approval was obtained by the UNSW HREC (approval number HC180864). All participants provided informed, written consent.

The entire sample of osteoarthritis patients (n = 26) who had been recruited from any of nine private practice physiotherapy clinics located across metropolitan Sydney were invited to take part in a semi-structured interview. These clinics were chosen due to their diverse geographical location across Sydney, each with a different socioeconomic profile, and associations with two of the research investigators (KM, JMN: who were not involved in the analysis of the data), as previously described [28]. The choice of these practices aimed to decrease selection bias as the practices were located in geographic areas of different socioeconomic status. The interviews occurred approximately six weeks after commencement of treatment. No therapist or clinic received any payments for their involvement in the study.

### Participant screening and eligibility criteria

Participants aged ≥ 45 years were eligible for the study if they met the following inclusion criteria: activity-related knee pain, morning stiffness lasting more than 30 min and had a clinical diagnosis of knee osteoarthritis as recommended by the NICE guidelines [6]. Exclusion criteria included: knee surgery in the last 12 months, hemi or total knee replacement of the affected joint, joint infection, inflammatory arthritis, gout, cognitive impairment, significant trauma, being treated under workers compensation [33], unable to understand English or unable to provide informed consent. Patients were screened by clinic reception staff when they called to book their appointment over a six-month period. They were re-screened by the consulting physiotherapist during their appointment and following verbal consent to participate in the study to the physiotherapist, eligible patients were provided with a reply-paid envelope which included a consent form. A researcher (RL) then contacted the participant to introduce herself, explain further details of the study and to arrange a time for a face-to-face (or telephone) interview.

Consenting participants completed a study proforma which included questions regarding sex, age, work status, height and weight (from which body mass index (BMI) was calculated), and medical history. The total number of people that were available from the larger study was n = 26, all of which were included, and consented, to participate in these interviews.

### Data collection

Patients’ experience and perspectives of the care received during their physiotherapy sessions and how their knee osteoarthritis was managed were gathered using semi-structured interviews either face-to-face or via telephone at six- weeks post-starting treatment. All interviews were conducted one-on-one by the same researcher (RL, female physiotherapist, trained by senior researchers in the team). These semi-structured interviews were primarily undertaken to explore patient perspectives and experiences of their physiotherapy care. The questions were designed by all authors with a range of expertise and experience across clinical physiotherapy, exercise physiology and health-service research and were anchored around the core first-line elements recommended in guideline-based osteoarthritis care (Supplementary information). Additional questions covered their perceptions and their satisfaction with their care, including on their understanding of information provided by the physiotherapists, if follow-up treatment plans were made, and how their care could be improved. Patient satisfaction with their physiotherapy care was also asked quantifiably within the interviews on a verbal numerical rating scale from 0 to 10 (with 10 being the most satisfied), as used previously [34].

Interviews lasted approximately 20 min and were held either face to face at the clinics (n = 23) or by telephone (n = 3) as required and were digitally audio-recorded with field notes taken by the researcher during the interview. The audio files were de-identified (RL) and transcribed verbatim by an external provider. The transcripts were not returned to the patients for correction, though each interview included the researcher checking with the patient iteratively with paraphrasing and confirming statements.

Transcripts were uploaded to NVivo software (v12). The transcripts were then coded using both content (alignment to guideline-based care and satisfaction with care) and thematic analysis approaches by researchers with an understanding of evidence-based osteoarthritis management [35]. The coding was initially inductively analysed separately by one researcher (RL) who discussed the codes with two other researchers (SD, JT) where the codes were further developed and revised. Content analysis explicitly investigated the references by the patients to elements of evidenced-based guideline management. The codes were also organised into broad themes that reflected experiences and perceptions of the patients of their physiotherapy care. Further refinement of the thematic analysis was undertaken by two researchers (HS, JT) who discussed and revised the coding tree with other researchers (SD, JN) and the final results were presented to the wider research team for discussion. To ensure rigour in the analysis, credibility – the research team were familiar with the management of osteoarthritis, dependability described above, confirmability – broad range of people in the research team including musculoskeletal experts and qualitative researchers and transferability – interviews continued until saturation had been achieved.

## Results

### Patient characteristics

Over a 6-month period, 26 patient participants consented and were interviewed for the study (11 males and 15 females) and they provided written informed consent. Fifteen physiotherapists, with an average of 10 years of practice (range 2–26 years), provided care for these patients across eight of the practices. The average age of the patient participants was 60 years (standard deviation: 9), 81% were white, 60% married with a mean of 15 years of education and 54% were overweight, mean BMI 26.7 (5.7) [28].

### Evidenced-based care provided

The patients described the varying extent to which physiotherapists are delivering evidenced-based management, as summarised in Table 1 and detailed below.

**Table 1:** Core or commonly recommended elements of treatment that patients stated that their physiotherapists were providing as per osteoarthritis guidelines*

Elements of Care	Content analysis	Sample quotes
Core or commonly recommended elements		
Prescription of appropriate exercise	26 (100%) patients said that they were prescribed land-based strengthening exercises to do at home.	*“So I’m very happy to try a series of strengthening techniques and just in the last couple of months that’s been very good for me… And plus the exercises seem to be working.” [Patient 3]*
Education about osteoarthritis	Three patients said their physiotherapist discussed information about osteoarthritis. 8 said that they had education not specific to osteoarthritis.	*“I guess he’s just provided an explanation of what’s going on with it to me. So I guess in that regard, he’s provided some education.” [Patient 6]* *“No. I have to say there was no real– just said, “Oh, you’ve got a bit of arthritis,“ or, “Yeah, we can manage that."“ [Patient 3]*
Education/Discussion about weight management	24 patients said that their weight was not discussed as part of their osteoarthritis care. 5 patients stated they knew weight was an issue.	*“He didn’t really concentrate on the weight thing.” [Patient 13]* *“if I can get the weight a little bit lower, that will help because that probably has an impact on the knee, and the more weight you’re carrying, the more pressure it’s putting on knees and other joints. So yeah, I’m aware of it…” [Patient 3]*
Non-core or secondary elements of care		
Education/Discussion about pharmacological management of pain and inflammation	6 patients mentioned that they had discussed medications with their physiotherapist	*“No, not really. I think it was probably mentioned but not so much in a discussion, just if you needed the anti-inflammatories… and I kind of knew that anyway… No he didn’t really go into detail with that.”* * [Patient 13]*
Referral for radiological investigation/imaging	Patients reported mixed responses to the need for imaging, which is not included in guideline care for osteoarthritis.	*“[Physio] didn’t want me to do it, necessarily do x-rays or MRI’s or whatever. Or going to see any specialist.”* *[Patient 3]*

* Guideline-based care references [4–8]

#### Prescription of appropriate exercise

Exercises were the most prescribed treatment by the physiotherapists. All participants said that they were prescribed exercises to do at home and they saw the benefit in doing the exercises to improve their knee pain.

*“Using the exercise routine that she gave me each week. And I could feel the progression. I could feel each exercise becoming easier than the one prior. The first time I tried it, and you could feel the strength coming back. From that, I could easily– and each week thereafter, it was just constant progression which I hadn’t ever experienced before. [Patient 14]*

The participants felt that the exercises made sense to them, were logical, and that they felt better for doing them and that the exercises were helpful. Examples of the type of exercises that the patients briefly discussed that they were prescribed are summarised in Table 2.

**Table 2:** Knee osteoarthritis patients’ recollection of the types of exercise that were prescribed by their physiotherapists

Exercise types recommended by Physiotherapist		Guideline recommendations (45)	Example quotes of patient’s description of exercise	% mentioned by osteoarthritis patients
Strengthening:	Sit to stands/squats	Strengthening exercises -strongly recommended	*“So one of them was squats, sitting squats, and up and down from a chair.” [Patient 13]]* *“wall sit with, what do you call those physiotherapy bands?” [Patient 15]*	58%
	Glute bridges		*“lie on my back, knees up and I lift my hip up” [Patient 19]*	23%
	Calf raises		*“standing on my toes” [Patient 17]* *“get on my tippy toes as high as I can” [Patient 15]*	27%
	Leg extensions		*“Get a towel, put under the hamstring and squeezing down” [Patient 10]*	15%
	Leg raises		*“lie flat on my back then I will bring that up and straighten it as best I can” [Patient 15]*	12%
	Leg press		*“using the machine. Pushing. Push, push, push away” [Patient 10]*	8%
	Hip abduction/ adduction		*“sitting on a chair with band around my knees, sitting on the edge of the chair and pushing the band out” [Patient 7]* *“using the band around my legs and sort of walking like a crab” [Patient 6]*	31%
Aerobic:	Aquatic exercise/ hydrotherapy	Conditional – dependent on patient preferences and accessibility	*“I just started swimming after I finish physio” [Patient 8]* *“She also suggested things like not running for one and not walking– at least earlier on she said to me don’t walk any great distances. Try not to use stairs. So to try and get, I think, the irritation to calm down.” [Patient 22]* *“I use a bicycle in the gym and I’ll ride that for about 15 min. And that seems to have improved those… muscles.” [Patient 5]* *“Gym work. Get on a bike. Swimming. Swimming and a bike, basically…” [Patient 24]* *“So I’ve got to do all those plus some bike to strengthen my quads” [Patient 6]*	27%
Stretches:		Conditional	*“use the roller… kind of on the – I think it’s the gluteus muscle”* *“…have this wedge. And I lean forward stretching the calf muscles at the back.” [Patient 26]*	35%
Balance:			*“and a lot of balance exercises… and the better balanced I can get, I’ll be able to walk a bit more comfortably.” [Patient 3]* *“it was a balancing one, which I do do as well on one leg to try and get the muscles to work around the knee.” [Patient 13]*	27%

*“… and as my knee got stronger, the exercises became more– changed and became more useful” [Patient 14]*

*“Balance. There’s balance. A lot of strengthening. It’s hard to describe. Strengthening from the tops of my legs to support the knee… Well, there’s so many. One on my back to help my back, lift your bottom. Lift your bottom. Oh, there’s a lot.” [Patient 18]*

Quadriceps strengthening was the most prescribed exercise type. The physiotherapists prescribed exercises that were mostly land-based muscle strengthening exercises targeting the lower limb muscles.

*“Yes, I was given just some exercises, basic, to help strengthen or keep the muscle strength around the knee up, because having the injury, obviously, the muscles start to deteriorate a little bit.” [Patient 20]*

Prescription of aerobic exercise was rarely mentioned by the patients and if it was, it was not clear if the patients had been doing aerobic exercise, e.g. swimming, cycling and walking, prior to seeing the physiotherapist or if it was prescribed to them. Some patients stated that their physiotherapist told them to do less aerobic exercises.

*“…and some restrictions on what I was doing, as well… But doing longer walk just was… not the thing to do.*” [Patient 21]

*“So I mean I went for jogs when she had told me not to go jogging, ‘cause my areas quite hilly uhh and doing more reps than I should of done, and some of those things. She said I didn’t ask you to do that, and I’m like I know, but I though it might help, so it went a bit back, because of that, it is more because of me, no because of her.” [Patient 15]*

Most of the patients said that they were pleased with the number (including frequency and duration), intensity, type and progression of the exercises that they were provided (summarized in Table 3). Patients briefly explained that their physiotherapist focused on only providing a few exercises for them to do and then progressed these when they improved. They also focused on the patients doing the exercises with the correct positioning and alignment.

**Table 3:** Knee osteoarthritis patients’ recollection of their physiotherapists’ general exercise recommendations that were prescribed to them

Exercise recommendations by Physiotherapist	Example quotes of patient’s description of exercise
Frequency of exercises: Number per session	*“[bridge holds] I do 2 repetitions of 6 [morning and afternoons]… the wall sit, and I’ll hold that for a minute, and I’ll do 5 of those…” [Patient 15]* *“so there’s eight of those times three [exercises]” [Patient 19]* *“I think if there’s too much that I wouldn’t have absorbed it enough. And if there’s too many exercises, I’m more than likely not to do them all because I just won’t have the time to do them all.” [Patient 4]*
Frequency of exercises: Number per day	*“The program they gave me, I liked it because it was just a few exercises to do twice a day sort of thing and done and dusted. So pretty manageable.” [Patient 5]* *“three groups of exercises to do each day or to do a couple times a day.” [Patient 16]* *“I’ve got my exercises that I have to do every day, twice a day” [Patient 4]*
Intensity of exercises	*“It’s hard work, but I got to do it.” [Patient 14]* *“that can get quite sort of difficult after a while” [Patient 22]* *“There’s been nothing too difficult…” [Patient 1]*
Duration of exercises	*“It takes and hour maybe an hour and a half with 30 min on the bike.” [Patient 8]* *“…night routine and that takes me about ½ an hour… morning is about 10 min” [Patient 15]*
Progression of exercises	*“three or four exercises each time I’ve visited, and it’s sort of progressed as I’ve visited as well in terms of being a little bit harder or more intense.” [Patient 7]* *“The exercises were extremely difficult on the first day at the session, but as the week went on, it got progressively easier. And then we progressed to the next exercise the following week, and so on and so forth, to the point where I had all the muscles woken up and was doing the exercises with little or no pain at all.” [Patient 14]*
Technique	*“I think she really focused on helping me correct my positioning of my knee over my big toe when I was squatting.” [Patient 19]*
Rest	*“One of them was rest and I didn’t do that [laughter] even though I was advised to. I continued to play football with it” [Patient 20]*

All patients said that the exercises prescribed to them to do at home were provided verbally, with most also stating that this was the only way information was given (“*all I needed*” Patient 15). However, for a few patients or if the patient asked, then they were provided with handouts (drawings “*Little stick figures he does*” [Patient 18], photos or written exercises) or they were sent an email or videoed doing the exercises on their phone. Some thought the other ways of providing of information, rather than just verbally, would be beneficial to assist in recalling the exercises.

*“It would be useful if I did have something in writing from the physio” [Patient 1]*

*“I think I understand them and you know she’s very good at demonstrating them and as I said she always emails them to me after the session… Because it’s all very well to think, “Oh, I’ll just remember all that.“ But, yeah. It’s weird how somehow you just can’t remember the fourth one.” [Patient 22]*

#### Education about osteoarthritis

When asked if they had received education from their physiotherapist regarding osteoarthritis, less than half of patients mentioned that they had received any type of education regarding osteoarthritis, with only three patients saying that the received specific education about osteoarthritis progression/pathophysiology. Although the education provided, or lack thereof appeared to be what the patients were expecting and at times was mostly more helpful than previous information that they had received.

*“Not really. Not education… But he said that, “You’re this thing because your joint is not good, because [cartilage?] is all gone. It won’t be back. It won’t be coming back. It won’t grow. But you have to manage what you have, ...“ But I asked that whether he saw patient like me because my specialist said that, “If you don’t get better, ultimate [goal?], you have to have a knee replacement.“ That was very worrying me. I was very upset with that.”* [Patient 8]

*“Yeah, she did. We talked about it quite a bit. I think the osteo– Well, we did talk about it a bit because I’d never heard– I mean I’d heard of osteoarthritis but I didn’t know what it was.”* [Patient 22]

Twenty five of the 26 patients interviewed reported that they had received enough general information about their treatment during their physiotherapy sessions, “*Yeah, she’s pretty thorough*” [Patient 1] although one patient felt that they were overloaded with information “*I was bombarded. Bombarded. It was too much. Too much*” [Patient 19].

*“I think because they’re constrained in time. Because during the time that they tried to treat me and they tell me and if they tell me, they will run out of time. Because they have only half-hour, 45 minutes so they don’t have time to tell too much. Even if you give me amount of information, I couldn’t digest it.” [Patient 10]*

#### Education/Discussion about weight management

When asked, patients seemed knowledgeable about the association between excess weight and harm to their knee joints, however few recalled that their physiotherapist had discussed weight management with them, and this was in spite of more than half of the patients being overweight or obese.

*“The physio hasn’t discussed that I’m fat [laughter].” [Patient 12]*

When weight management was discussed, it may have been framed around other issues, with not many physiotherapists clearly linking excess weight to the condition.

*“She did talk about that in that I asked her I guess well, I don’t know if I asked but when we looked at the MRI report and it had mention of osteoarthritis, and I got her to explain that more to me. Because as I said earlier, I didn’t know anything about arthritis and she said one of the management factors for osteoarthritis is weight control or maintenance or whatever you call it, correct weight... once people get overweight, it exacerbates the osteo quite a lot or it can.” [Patient 22]*

Patients understood the theory behind losing weight to relieve their symptoms.

*“if I can get the weight a little bit lower, that will help because that probably has an impact on the knee, and the more weight you’re carrying, the more pressure it’s putting on knees and other joints. So yeah, I’m aware of it, but I’ve just got to get it a little bit– get my weight down a couple more kilos, I’ll be happy.” [Patient 3]*

Five patients mentioned that they already knew that losing weight was important, even if this had not been discussed by their physiotherapist.

*“No, we haven’t yet. But I do know that that can be an issue” [Patient 18]*

*“I actually asked the question myself [re: weight]… But the critical thing is getting the muscles around fixed, and turned on, and built up.” [Patient 14]*

Several patients thought that weight had not been mentioned as they did not perceive themselves as overweight.

*“And not with the knee specialist, either, because I was 55.8 kilos. And he said, “We don’t worry about your weight. You’re right.“” [Patient 17]*

Five other patients mentioned that their doctor had discussed weight in relation to their osteoarthritis, though only one patient mentioned that their physiotherapist had weighed them. Only one patient reported receiving direct advice regarding weight loss and no one said that they had been referred to a dietitian, although one said that they had been “*referred by a nutritionist*” [Patient 19] to the physiotherapist who they were seeing regarding menopause. One patient did not feel that their GP was very helpful.

*“I guess I think it’s just if you have osteo, you have osteo. There’s not much I can do to not have it. I definitely think probably weight has a lot to– it’s not good. I know that’s not good. I’d like dieticians to be more available… I have been to a dietitian in the past. Suggested to my GP I’d like to go to a dietitian, and she said, “What for? You know what to do.“” [Patient 24]*

#### Pharmacological management

the patients said that they would go to their GP for pharmacological management. Six of the participants briefly mentioned that they had discussed medications for their knees, with seven other patients mentioning that they had had a discussion with their doctor but not their physiotherapist.

*“It’s something my doc would have told me anyway. I probably would listen to my doctor more… on medication”* [Patient 2]

A few patients mentioned that they took other medications like “*Glucosamine, yes. I’ve been taking that for a while. I don’t think I’ve even mentioned it to [Physiotherapist]*.” [Patient 1].

*“So for a number of years, I’ve been taking 6,000 milligrams of fish oil and 3,000 of glucosamine. Whether it’s a placebo or not, I don’t know. I feel better when I’m taking it.” [Patient 11]*

#### Referrals

Most patients did not recall any referral for knee investigations by their physiotherapist to other health professionals and none were referred to other services such as exercise physiology, mental health advice or for other specialist services e.g. Arthritis Australia. One patient had self-referred to a dietitian in the past.

*“I’ve done that on my own bat and gone and seen a dietitian. I’ve done all of that. I wasn’t overly impressed, so I’m just managing my own… My biggest dilemma is the movement, so I need to get that sorted so that things will shift.” [Patient 4]*

There were mixed responses to the need for imaging, which is not included in guideline care for osteoarthritis. However, there did not seem to be clear reasons provided by the physiotherapists as to why imaging was not necessary. Three patients did say that their physiotherapist stated that they did not need to look at images, six patients mentioned they had images already and only one said the Physiotherapist required scans prior to their appointment. Some patients went to get images whether their physiotherapists advised them to or not.

*“No. Although [Physio] did suggest that I get an ultrasound. I then went to my GP to get a referral for that. And the GP suggested that I have an MRI instead, which I did do… I do think that has been beneficial because it’s given everybody, I think, a lot more information about what is going on with my knee.”* [Patient 22]

*“[Physio] didn’t want me to do it, necessarily do x-rays or MRI’s or whatever. Or going to see any specialist. He said, “I think it’s just wear and tear. And it’s something you can manage.“ I have actually got an x-ray done as a sort of second check on that. And I might even see a knee specialist too.”* [Patient 3]

Most participants had actively sought care and self-referred to physiotherapy for management of their knee pain, with some referred to physiotherapists from their GP or specialist. Some recalled that the process of referral between health professionals was not a simple one.

*“I spoke to my doctor about it, and he said, “Well, let’s get the x-rays,” and then he said, “Go to your own physio.” [Patient 1]*

*“I already seen a specialist before I saw my physio. My specialist told me, “You need physio”* [Patient 8]

*“I was actually referred from my GP to a sports physician to the physio. So, I guess I came through a lot of referrals rather than the other way around.”* [Patient 11]

Only a couple of patients had been asked to see a specialist or GP regarding their osteoarthritis by their physiotherapist.

### Satisfaction of care

The patients rated their satisfaction of their physiotherapy care as high (mean 8.3 (1.6)); the patients’ ratings ranged from a “*3 to 4*” out of 10 *(“I mean I haven’t used it… We just didn’t click”* [Patient 19]), though 21 of the 26 patients rated 8 or above (“*I think that I’ve had the best*.” [Patient 17] and *“I’d say that it would have to be at least a 9 out of 10. I mean, it’s been a considerable benefit.”* [Patient 5]). Their satisfaction with care appeared to link closely with their improvements following the prescribed exercises and their positive therapeutic relationship with their physiotherapist (see below).

Several patients also mentioned other strategies the physiotherapists advised, such as icing, and taping/brace on the knee and those that had these thought that they were beneficial:

*“I’ve got a physical job, so their taping of [my knee] really helped.” [Patient 20]*

*“Icing, when I had some localised swelling”[Patient 23]*

*“But since he strapped the knees, it has been easier to do it” [Patient 26]*

*“They put the ice maker around me [laughter]. Yeah. So they iced it more. That was a fair amount of the treatment.” And “But I think rest, initially, with my knee, helped a great deal.” [Patient 16]*

*“I guess massage manipulation… there was a lot of work on relieving the pain of the knee cap, which is fantastic, and that made a big difference.” [Patient 24]*

In contrast, one patient stated that:

“*I found nothing, absolutely nothing useful because it doesn’t matter how much acupuncture or dry needle, whatever he calls it he does, how much massaging he does, I walk out of there fine. Two days later I’m back with it again. The swelling hasn’t gone anywhere.” [Patient 12]*

### Themes relating to patient perceptions of care

The analysis of the data identified five major themes that highlight the importance of the positive therapeutic relationship between patient and physiotherapist that enable patients to engage with the process and achieve satisfactory results. The patients also were able to provide an insight into how treatment could be improved. The main themes are summarized in Table 4 and detailed below.

**Table 4:** Thematic analysis of knee osteoarthritis patients’ perspectives of their private practice physiotherapy treatment

Themes	Constructs	Sample quotes
Patients are keen to engage with the process to relieve pain and remain active	Relieving pain is their main concern and was the main reason for seeking physiotherapy care and they were keen to engage in their treatment. Patients said their symptoms were ‘not that bad’, that they wanted to stay active and to last as long as they can without an operation. Patients wanted guidance through the process of pain relief through exercises and came to the realisation they would need to work at it.	*We really haven’t spoken about the osteoarthritis side of it. That’s my biggest complaint, the ligaments and the pain, so we are dealing with that to start with.” [Patient 15]* *“I want to give this a real solid go with the exercises.” [Patient 3]* *“I’m very aware of– I feel like I’m on borrowed time with my knee. That sounds really weird. I want to do everything now to build up that quad muscle to keep my knee supported.” [Patient 19]* *“And that’s when I got into the physio side because I refused the operation.” [Patient 12]*
Importance of communication and connection with their physiotherapist	The patients appreciated that their physiotherapists were approachable, open, personable and committed, who actively listened and were supportive. Patients needed to connect with their physiotherapist to maintain their treatment. The physiotherapists were able to alleviate patient concerns, provide realistic expectations and discuss solutions.	*“I’m happy with X and what she does and how she explains things. And she tries to come up with different solutions.” [Patient 11]* *“I understand the goals of the treatment and why it’s a good idea. Given the… the tracking issue was identified about 20 years ago. I’m not sure how we’ll go correcting the sins of a lifetime. And the genetic deformation sort of makes it interesting. But I understand the goal. And hopefully, it will slow down any further wear and tear I guess.” [Patient 11]*
Physiotherapists focused on their treatment only, not their osteoarthritis	Some physiotherapists’ lacked clarity in their understanding of osteoarthritis, which included coming across as knowing too much or not enough about osteoarthritis. Patients do not expect physiotherapists to have time to discuss other aspects of osteoarthritis treatment	*“I just felt she was being very diagnostic within the half an hour… but she’s saying, “Oh, I don’t think you have a problem. I think you just need to strengthen up your quad a bit. You know, it’s not that bad.” And it was all very—not that she was sort of dismissing it, but it was like, “Oh, it’s just your knee.” … and I’m wondering if they actually do know what they’re talking about?”* * [Patient 19]*
Treatment works / is effective	The prescribed exercises, focus on strengthening/supporting the muscles around the knee, are logical and they do work. Other aspects of treatment that is more ‘hands-on’ is now less frequently prescribed, e.g. taping and massage, though were thought to be beneficial and desired by some patients.	*“I think in terms of self-management, I think they were quite helpful. They’ve certainly understood how much I could feasibly do or bear, given the problem and didn’t overload me with unreal expectations. So, I think it was pretty spot on” [Patient 7].* *[about previous Physiotherapist] “And I’m thinking, “If you can get to the nitty-gritty of massage… rather than trying to explain.“ Where I find him a lot better. He’s more hands-on and does the treatment while I’m in there.” [Patient 2]*
How treatment could be improved	Exercises need to fit into their daily routine as patients can forget them and more are likely to stop when they feel better. Exercises were explained well at the time, mainly verbally, but patients can forget them, so other non-verbal information would be useful. Patients want more education of osteoarthritis and a simpler referral system. Patients want an ongoing management plan and advice and to know when their sessions will end. There is a fine line between too many sessions and not enough: price is a factor for continuing treatment. Patients would like group classes for social aspect and better education of osteoarthritis, though 1 to 1 treatment is thought to be better for ‘tracking of knee’ and ‘hands on’ treatment.	*“I mean I know I don’t do the exercises, but I do know that they make sense, and I can understand the logic behind them.” [Patient 13]* *“Yes, it’s just a matter of doing them, isn’t it? I mean, yeah, we all do it. But making the time– I mean, I tend to do– if the knee’s going along okay, I tend to not, which is wrong.” [Patient 13]* *“I don’t remember what she said but she explained it very well.” [Patient 22]* *“Yes. Sometimes I’ve got to think exactly what he said [laughter] they [exercises] were” [Patient 13]* *“And I suppose what’s the missing element is that longer-term management, ongoing management, and probably even a clear diagnosis. Really knowing what it is that I’m suffering sort of thing.” [Patient 7]* *“Just a solid answer of what can be done. I don’t want to spend $75 every time I go to the physio. I don’t want to have to do that for the rest of my life… So a long-term solution. And as I said, I just can’t afford to be paying that sort of money every week for the rest of me life.” [Patient 12]* *“Look, group session would more than likely be cost effective, i.e., cheaper. And I don’t want to put it all down to cost, but I’d be more than happy to go into a group session for that.” [Patient 7]* *“Hard to say isn’t it [Group vs individual session]? The doctor seems to think it’s the tracking of the knee, so on a group basis, would they be able to work on the tracking on the knee?… A group thing would be fine. I wouldn’t really matter too much to me as long as it’s helping.” [Patient 26]*

### Theme: patients are keen to engage with the process to relieve pain and remain active

The patients that sought private practice physiotherapy care went to get relief of their pain, but often expressed that their symptoms were “*not that bad*”. Though they did believe that they were on borrowed time with their knee joints. The participants were the type of people that enjoyed a range of exercises and sports and keen to engage in the treatment. Patients stated that they wanted to stay active and “*keep what you’ve got for as long as you can” [Patient 3]*, hoping to avoid knee surgery.

*“I’m just hoping that I get more mobility and less pain overall. That’s my main aim of coming… so that I can go a few more years without having to be operated”* [Patient 4]

They wanted guidance from the physiotherapists and answers to their questions about what they could do to relieve their symptoms. They found coming to their physiotherapist was useful in terms of lessening their knee pain and to have a greater understanding that their pain could decrease. Although the patients realised that they would have to put work in to ensure that this occurred (“*Puts the responsibility on me.”* [Patient 15]).

*“I’ve now gained a lot more information about what’s wrong with the knee and a lot more insight into, I guess, what to expect.”* [Patient 22]

*“She’s the expert… It’s hard work, but I got to do it… Yeah. I was quite amazed. Because I thought I was going to be living with this pain for the rest of my life… I figured this was just a part of getting old. But truly it’s not the case.”* [Patient 14]

The patients also said that they did not need or want to take medications.

*“So I don’t take pain relief if I don’t need it. I’m one of those I don’t like just to pop pills.” [Patient 25]*

### Theme: importance of communication and connection with their physiotherapist

The patients described their physiotherapist to be approachable, encouraging and committed (“*very open and very helpful*” [Patient 10]). They appreciated the attention from the physiotherapists from having someone who is “…*very personable, very easy to talk to*” [Patient 5] as well as providing a solution by discussion their concerns. The ability to communicate well seems to have influenced the patients’ positive satisfaction with the care that they received. All patients reported being able to ask the physiotherapists questions and most felt comfortable in doing so. Thus, they were able to clarify their understanding “*I could ask as many questions as I want*” [Patient 15], “*Yeah. Very open*” [Patient 13]. Moreover, all but one patient thought that the physiotherapists were able to answer their questions to their satisfaction.

*“I think she’s a good communicator and had answers to my questions and is approachable. And so I feel like I can ask her anything I like.” [Patient 22]*

A patient stated that if they did not connect well with their physiotherapist, then they switched to another or did not come back.

*“I started with one physio where I was overloaded with information, and that was too much. And then I went back to my original physio and absolutely, yeah. I mean it was really well explained.” [Patient 19]*

Patients appreciated that their physiotherapist was able to alleviate their concerns that they had regarding that their knees would not get better. Patients became more positive regarding the possibility that they can improve and not to lose hope after seeing their physiotherapist. However, they also now had a greater realization that their knee/s were not going to be the same as before, that they need to manage their movements and to do regular exercises.

*“I guess the fact that I’m getting someone to actually help me through the process rather than try and deal with it myself… I think that’s been useful.*” [Patient 1]

### Theme: physiotherapists focused on their treatment only, not their osteoarthritis

The patients believed that the physiotherapists were experts in treating their symptoms, which is what they said the physiotherapists focused their treatment on. However, some thought that their physiotherapist lacked clarity in their understanding of and diagnosis of their osteoarthritis. Several patients mentioned that their physiotherapists described their condition as being a result of “wear and tear”. Some patients did not know that they had osteoarthritis.

*“I didn’t realize it was wear and tear. Because I’d never had anything to do with arthritis, I think what I was really thinking about was what would be called rheumatoid arthritis. So that’s why I was very—everything she was telling me was pretty new. She gave me enough information is what I’m trying to say I think.” [Patient 22]*

Some patients thought that their physiotherapist came across as not knowing enough about osteoarthritis whilst other said that they came across as knowing too much. One patient mentioned that the physiotherapist “*could read more into the report or explain it better than the GP did, I thought*” [Patient 22] (see Table 4).

*“I just wanted to have a bit of base knowledge on top of the physio’s sort of gut feeling that you haven’t got —I think he felt my knees were in reasonable sort of condition. But it’s nice to know what the facts are. So I’ll have a think about whether I go and do some extra investigations.” [Patient 3]*

Patients did not expect that their physiotherapist would have much time to discuss other aspects of osteoarthritis treatment, this included pharmacological and weight management, and referrals to other health professions. They were not provided information about professional osteoarthritis organizations and were provided with mixed messages regarding the need for imaging (see above).

*“No. They did not mention much about that. Yeah. And I think because they’re constrained in time.”* [Patient 10].

### Theme: the treatment works / is effective

Patients felt that the exercises prescribed by their physiotherapists and the self-management strategies were appropriate and helpful.

*“I think the exercises she’s given me have helped, and I think that the fact that she has reviewed them and changed them as my knee has progressed” [Patient 22]*

Of the patients that had been to a physiotherapist previously, they thought that physiotherapist treatment had changed over time; with there being less of a ‘hands-on’ focus now. They did still expect some ‘hands-on’ treatment by their physiotherapist, such as massage, which some patients preferred to just being instructed to do exercises.

*“I would say the exercises that I do away from the clinic [were the most useful]. But then I would have to say the massages she gives me are quite good.” [Patient 15]*

### Theme: how treatment could be improved

The patients thought that the exercises needed to fit into their daily routine, otherwise they were more likely to forget to do them. A few patients mentioned that they were either overloaded with exercises or did not do all the exercises that were prescribed. Some became ‘lazier’ over time or found it harder to keep trying to fit the exercises into their daily routine. Patients were also likely to stop doing the exercises when their symptoms improved or if the exercises were painful or “*when I feel I need to.” [Patient 16]*. In addition, many patients became hesitant and/or vague on the exact details of the exercises and could not give a clear name of the exercises. However, they could often describe how to do the exercises clearly.

*“Sometimes I’ve got to think exactly what he said [laughter] they were. One of them was squats sitting… he felt that my muscle wastage in my thigh was considerable... To tell you the truth I don’t do them every day… What was the other one? I’ve got them written down somewhere. There was a third one as well… The whole idea was just to strengthen those muscles so it could support it a bit better.” [Patient 13]*

*“Well, one, I’m lazy. And two [laughter], if something hurts I don’t do it. And the exercises that he’s giving me hurt.” [Patient 12]*

The patients believed that the exercises prescribed by their physiotherapists were explained well during their consultation, but they did reflect that they often forgot some of the exercises. Patients’ recall and understanding of the management they were given by their Physiotherapist for their knee osteoarthritis varied, however, all patients interviewed were able to recall at least one aspect of the recommended guidelines being included in the management of their knee osteoarthritis. Some patients recalled their exercises easily and were doing them regularly, though, when asked to elaborate many were hesitant or unable to provide details about what they should be doing.

*“The only thing that would be helpful for me is sometimes I tend to forget what I’m meant to be doing and sometimes when you’re shown an exercise, you may not fully remember exactly how to do it. So even if there was like videos that you could watch or just sheets sort of describing the exercise or just notes on which ones you’re meant to be doing and how many times a day and things like that because yeah, it was just all verbally passed on to me and sometimes you might remember—” [Patient 6]*

The patients said that they would prefer to have had more detailed education regarding osteoarthritis and have an ongoing management plan of how to live with osteoarthritis. This, included knowing when their sessions with the physiotherapist would end.

*“… I probably would have liked to have heard the word arthritis used. Or a little bit more of an explanation of about how it comes about and what things to avoid or do more of.... I probably would have liked a little bit of more long-term advice or awareness that, given this is osteoarthritis or whatever, you’re probably going to have to live with it for the rest of your life, and here’s how we suggest you… manage it. Something along those lines.” [Patient 7]*

In contrast, there were a few patients that questioned if physiotherapy was helping their knee and they thought that there was a fine line between too many sessions and not enough. The cost of the sessions was a factor for continuing treatment for some patients.

*“And that’s the dilemma I’m in is do I stop, or do I keep going? Because it’s not getting any better. I can go there every day, and I ain’t getting any better. So, do I stop and save the money and put it towards a knee replacement?” [Patient 12]*

Some patients stated that they would like to have group classes with other osteoarthritis patients for the social aspect, the cost benefit and to gain a better education of the condition. However, they felt that one to one treatment was better for maintaining correct tracking of the knee, the hands-on treatment and the ability to ask specific questions relating to their condition.

*“I think if I felt the group session was beneficial, I would attend a group session to maybe try to see how other people, how they’re coping, and see if there’s any other suggestions that come from group sessions. So sometimes people can, “Well, I do this and I find this rhythm helps.“ It’s a communication with people that are suffering the same problem.” [Patient 16]*

## Discussion

The description by knee osteoarthritis patients of their physiotherapy care that they received aligns with the evidence-based guideline recommendations predominantly for providing exercises, especially strengthening of muscles around the joint. However, other guideline recommendations were included less often or inconsistently. There was less emphasis by the physiotherapists on weight management, non-strengthening exercise prescription and education about the condition. The current study has also provided insight into how people with knee osteoarthritis perceive their physiotherapy care. People sought physiotherapy care for pain management and to stay active, and they were prepared to do their prescribed exercises as they deemed them beneficial. From the patient’s perspective, good communication and understanding from their physiotherapists was important to enable them to engage with the process and achieve positive results. They felt that physiotherapists are providing the type of care that they expected, and they were pleased with how their symptoms had improved.

Although exercise management is one of the key guideline-based recommendations for knee osteoarthritis, evidence has demonstrated that the combination of exercise with the other core aspects of first-line care, such as weight management and education of the condition, may enhance patient outcomes over exercise alone [4, 6–8, 11, 36]. Previously, Australian physiotherapists have reported focusing on the biomedical assessment of their patients via short-term goal-orientated exercise with consideration of other aspects of guideline-based care such as weight management advice being outside their professional role [27]. Similar findings from UK physiotherapists in 2008 also highlight the attention on strengthening exercises over aerobic exercises with limited follow-up services provided [26]. The current patients’ perceptions of their care, together with our audit of what was provided [28], would confer that these exercise-focused management approaches are still evident in Australian private practice, perhaps to the detriment of improved knee osteoarthritis patient overall outcomes.

Osteoarthritis patients continue to have anatomical-centred beliefs about their condition, which may in part be reinforced by their physiotherapists as highlighted in the current study with terminology such as ‘wear and tear’ still being used in practice. This has also been observed in people with knee osteoarthritis recruited from general practices, community physiotherapy clinics and the community [30]. Although, the participants in the current study, who were attending primary care physiotherapists, did seem to be more positive than previous studies regarding their attitudes to exercise and the fact they felt that surgery was not inevitable.

There is growing evidence that treating knee osteoarthritis holistically is more beneficial than as a biomechanical disease: for example, the combination of exercise and patient education has been observed to be superior to education alone [37]. The current patients highlighted their desire for more education, especially regarding their osteoarthritis as they conveyed that their physiotherapists focused on manual/physical treatment not the disease. Although the osteoarthritis education provided may not have been aligned with current evidence-base practice, if provided at all, the type and amount of education did appear to be what the patients were expecting; and at times was mostly more helpful than previous information that they had received. Guideline-based treatment includes education combined with exercise therapy to enhance improvements in function, pain and adherence [11, 37, 38]. Treatment should also cover a range of modifiable osteoarthritis risk factors individualised and developed in accordance with patient preferences and habits [36].

The current patients felt positive following their physiotherapy treatment about their knee osteoarthritis outcomes as they could see that their prescribed exercises were beneficial. Through their treatment they realised that they had to put work in themselves to attain their goals, but the physiotherapists provided confidence to them to continue to exercise. Patients mostly complied with the exercises when they saw benefits. Using this positive relationship, physiotherapists have an excellent opportunity to provide more osteoarthritis education and long-term management within their treatment. The current patients also said that they would likely attend group classes, for decreasing the cost of care and to meet and discuss aspects of the condition with others under the guidance of their physiotherapists. Thus, interventions such as Good Life with osteoArthritis in Denmark (GLA:D) that include evidence-based education and supervised exercise delivered predominantly by physiotherapists may be likely to be acceptable in this patient group [11, 39]. Also, provision of more than a verbal description of exercises to patients would be beneficial to maintain adherence.

Patients with knee osteoarthritis in the present study found their exercise prescription to be effective. Whether the addition of more holistic exercise prescription, including aerobic exercises that may assist in the prevention and/or treatment of other chronic conditions as well as weight management and incorporation of other aspects of guideline care, is still to be determined. Interestingly, patients did not believe that some of the other aspects were part of physiotherapists’ roles, and some would like their physiotherapists to still focus on the one-to-one care, including taping and massaging (conditional guideline recommendations), within the limited time they had.

Few of the current knee osteoarthritis patients recalled that their physiotherapist had discussed weight management with them, even though more than half of the patients were overweight or obese. People who are overweight or obese are at greater risk of developing knee osteoarthritis, with evidence of weight loss being an effective treatment to relieve pain and improve function [36]. However, physiotherapists are currently not employing weight loss management enough in routine clinical practice [16, 28]. The patients in the current study did seem to understand the relationship between weight management and knee pain. When their physiotherapists did discuss the combination of losing weight and strengthening the muscles around the joint, this was well received by the patients. Thus, if physiotherapists were able to incorporate broader positive lifestyle change education, covering physical activity, weight management and psychological factors, this may improve patient outcomes further [16, 40, 41].

Although the present study was able to explore current perceptions of physiotherapy care for knee osteoarthritis patients, qualitative research is not designed to be able to generalise these findings to a larger population. The study was conducted across private physiotherapy clinics in Sydney. Although the clinics were chosen to represent a range of socioeconomic groups, being confined to only private practice patients, the views of the participants may be more representative of a more highly educated group patients that can afford private practice costs. It is also unknown as to whether the practices that agreed to be included in the study may have a greater understanding of and interest in providing guideline-based care than that of other practices. The semi-structured interview questions focused on the elements of osteoarthritis non-surgical guideline-based care, and as such, some of the other aspects of their physiotherapy treatment were not explored in more detail.

A key future target would be to focus on how to develop strategies to implement all the core evidence-based guidelines to improve knee osteoarthritis patient outcomes into sustainable physiotherapy implementation, that prioritises optimal care with patient expectations and satisfaction of their care. The development of resources and training for healthcare professionals delivering osteoarthritis care are a priority action [42], with strategies being explored to bridge the evidence-to-practice gaps [43].

## Conclusions

Knee osteoarthritis patients engaged with the treatment provided by their primary care physiotherapists to assist them manage their pain and remain active. Their positive perceptions appear to be mainly driven by the strength-related exercise component of care and is despite perceived limitations in the educational components of guideline-base care and what format the exercise programs are provided. Despite some perceived shortfalls in guideline-based care, patients do appear to be satisfied and appreciated the positive therapeutic relationship with their physiotherapists. However, it may be possible to improve patient outcomes further if more guideline-base care elements are regularly provided by their physiotherapists, including enhancing osteoarthritis education and long-term disease management, which patients would appreciate within their treatment.

## Electronic supplementary material

Below is the link to the electronic supplementary material.

Supplementary Material 1

## Data Availability

The datasets used and/or analysed during the current study are available from the corresponding author on reasonable request.
