# Supplementary material for: Knee osteoarthritis patient perspectives of their care in an australian private physiotherapy setting: a qualitative exploratory interview study
Source: BMC Musculoskelet Disord. 2023 Jul 11;24:564. doi: 10.1186/s12891-023-06692-4 (PMC10334626; doi:10.1186/s12891-023-06692-4)
Supplement: Supplementary file 1 — Supplementary Material 1 [file 12891_2023_6692_MOESM1_ESM.docx]

**Supplementary information: Participant interview transcript**

Q1. Has your physiotherapist discussed self -management of your osteoarthritic knee with you?

Q2. Has your physiotherapist offered education about your osteoarthritic knee?

Q3. Has your physiotherapist discussed exercise in relation to your osteoarthritic knee with you?

Q4. We know that sometimes people can find managing and living with arthritis negatively affects their mental or psychological health. Has your physiotherapist discussed this with you?

Q5. Has your physiotherapist discussed pharmacological (drug) management in relation to your osteoarthritic knee?

Q6. If BMI is indicated as over 25: Has your physiotherapist discussed weight management in relation to your osteoarthritic knee with you?

Q7. Did you understand all the information you were provided? How was this information provided? (Websites, printed, verbal etc)

Q8. Do you think you are able to implement the suggestions made by your physiotherapist?

Q9. Did you feel you ask your physiotherapists questions? Were your questions answered to your satisfaction?

Q10. Has your physiotherapists made a follow up treatment plan with you?

Q11. Are you aware of any healthcare funds you can access?

Q 12. Would you prefer a one to one session for you knee or a group session with other people who have the same diagnosis?

Q13. How do you rate your level of satisfaction with the care management of your osteoarthritic knee, on a scale of 1-10?

Q14. How did you feel about using the patient reported outcome measure form?

Q15. Can you describe what you found useful in the management of your OA knee?

Q16. Do you have any suggestions on how your care could be improved in the future?
